# Higher very short-term blood pressure variability is associated with lower atrial fibrillation recurrence after catheter ablation

**DOI:** 10.3389/fcvm.2026.1779540

**Published:** 2026-03-16

**Authors:** Sadahiro Murota, Minoru Nodera, Naoto Ohashi, Ryo Ogawara, Yu Sato, Tetsuro Yokokawa, Tomofumi Misaka, Shinya Yamada, Takashi Kaneshiro, Masayoshi Oikawa, Akiomi Yoshihisa, Yasuchika Takeishi

**Affiliations:** Department of Cardiovascular Medicine, Fukushima Medical University, Fukushima, Japan

**Keywords:** atrial fibrillation, baroreflex sensitivity, blood pressure variability, catheter ablation, pulse transit time

## Abstract

**Background:**

Blood pressure variability (BPV) is a cardiovascular risk marker independent of mean blood pressure. Prior studies have suggested potential associations between BPV and atrial fibrillation (AF) recurrence, but the relevance of very short-term, beat-to-beat BPV remains uncertain.

**Methods:**

In this single-center retrospective cohort study, we enrolled 153 consecutive patients undergoing first-time AF ablation who underwent pre-procedural overnight polygraphy with simultaneous pulse transit time-based beat-to-beat blood pressure monitoring. Very short-term BPV was quantified as the standard deviation (SD) of nighttime beat-to-beat systolic, diastolic, and mean blood pressure (SBP, DBP, and MBP); patients were dichotomized at the median. The primary endpoint was the first recurrence of AF lasting ≥ 30s beyond a 3-month blanking period during 12 months of follow-up.

**Results:**

During follow-up, AF recurrence occurred in 15 patients (9.8%). Kaplan–Meier curves showed significantly lower recurrence rates in the higher BPV groups (SD of SBP: *P* = 0.015; SD of DBP: *P* = 0.002; and SD of MBP: *P* = 0.003). In univariable Cox proportional hazards models, higher BPV was associated with lower recurrence risk (SD of SBP: hazard ratio 0.519, 95% confidence interval 0.311–0.865; SD of DBP: hazard ratio 0.258, 95% confidence interval 0.101–0.664; and SD of MBP: hazard ratio 0.251, 95% confidence interval 0.099–0.638).

**Conclusion:**

Higher nighttime very short-term BPV was associated with lower AF recurrence after first-time ablation. Pulse transit time-based cuffless monitoring may provide a practical method for preprocedural risk stratification; confirmation via multicenter studies is warranted.

## Introduction

Atrial fibrillation (AF) is the most common sustained arrhythmia, and is associated with increased risk of stroke, heart failure, and all-cause mortality (1, 2). Catheter ablation is an established AF therapy that improves rhythm control and may reduce cardiovascular events and mortality (3, 4). Despite advances in ablation techniques that have lowered recurrence rates (5, 6), a substantial proportion of patients still experience recurrence, making accurate prediction a critical clinical issue (5, 7).

Several predictors of AF recurrence after ablation have been reported, including sex, AF type, biomarkers, and imaging parameters such as left atrial diameter and atrial fibrosis (8–11). However, the predictive accuracy of these conventional markers remains relatively modest, highlighting the need for novel predictors.

Recently, blood pressure variability (BPV) has emerged as a cardiovascular risk factor independent of mean blood pressure (BP) (12). Increased short- to long-term BPV, assessed via home, office, or 24-h ambulatory monitoring, is associated with incident AF or post-ablation recurrence (13–16). In contrast, very short-term BPV, which reflects beat-to-beat fluctuations, has not been investigated in this context.

Pulse transit time (PTT), defined as the interval between the electrocardiographic R-wave and the arrival of the peripheral pulse wave, allows cuffless BP measurement and continuous beat-to-beat monitoring (17). The PTT-based system used in this study is based on a previously evaluated proprietary algorithm for cuffless BP estimation (17). Using the same algorithm platform, we have applied PTT-derived very short-term BPV measures in patients with sleep-disordered breathing, heart failure, and ischemic heart disease and demonstrated associations with adverse cardiovascular outcomes (18–20). However, this approach has not been specifically investigated in patients with AF.

Therefore, the present study aimed to investigate the association between very short-term BPV, assessed via PTT-based beat-to-beat monitoring, and AF recurrence after first-time catheter ablation.

## Materials and methods

### Study population and design

This was a single-center registry-based retrospective cohort study conducted at Fukushima Medical University Hospital. At our institution, an institutional registry systematically collects clinical information and examination data from patients with cardiovascular diseases. Between April 2018 and March 2024, patients with cardiovascular diseases were registered in this institutional registry.

Among patients registered during this period, those admitted for catheter ablation for AF were consecutively identified. Sleep-disordered breathing (SDB) has been recognized as a cardiovascular risk factor (21). Because SDB is highly prevalent in patients with AF and evaluation of nocturnal respiratory and circulatory status is clinically important (22, 23), overnight polygraphy was performed as part of routine clinical care during hospitalization when clinically indicated. When overnight polygraphy was performed, PTT–based beat-to-beat BP monitoring using the same device was conducted simultaneously to obtain nighttime BP data. These overnight recordings were obtained on the night before catheter ablation.

**Figure 1:**
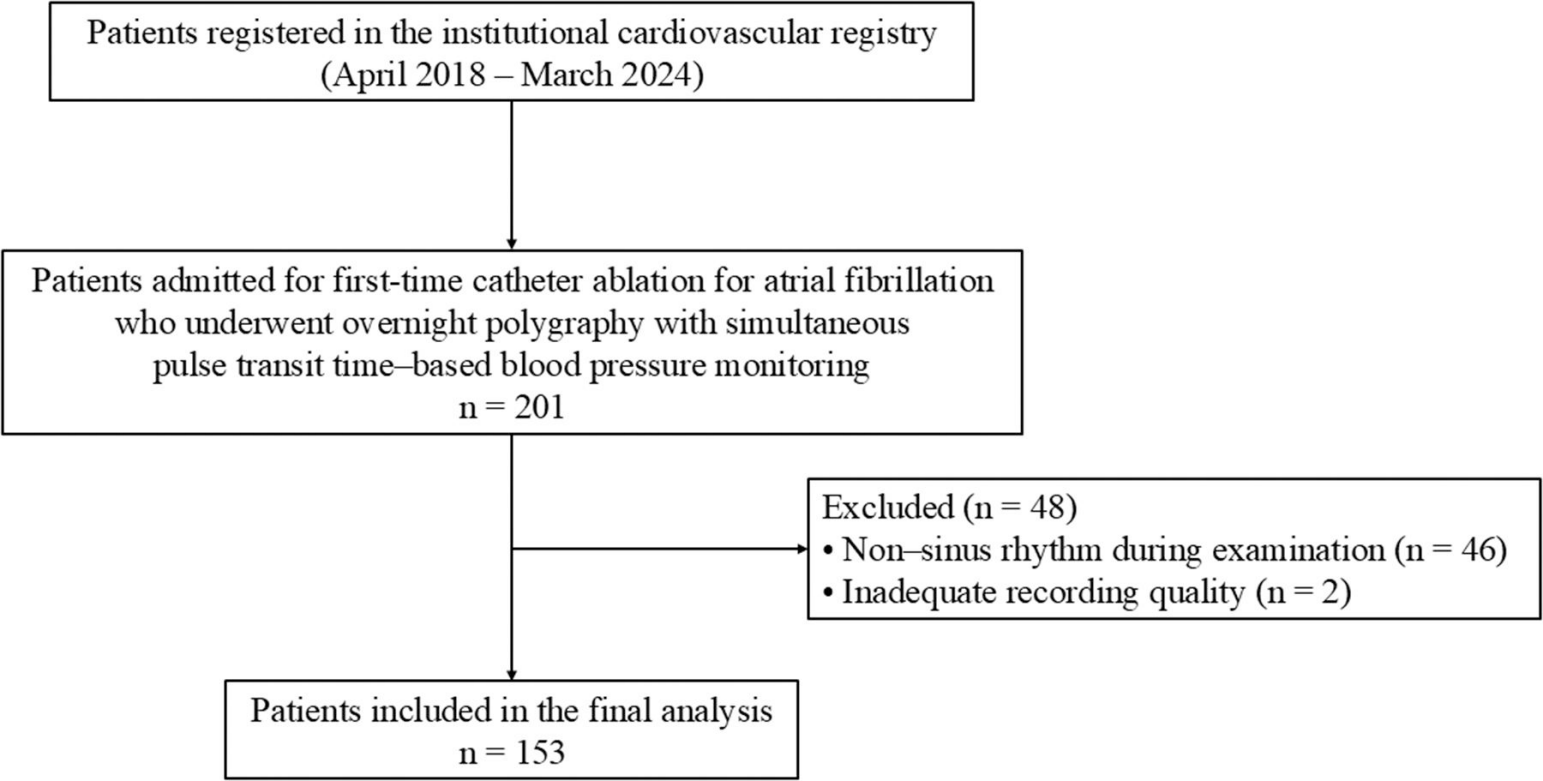
Study flow diagram of patient selection. In the institutional cardiovascular registry between April 2018 and March 2024, 201 consecutive patients admitted for first-time catheter ablation for atrial fibrillation who underwent overnight polygraphy with simultaneous pulse transit time–based blood pressure monitoring were identified. Forty-eight patients were excluded (non–sinus rhythm during the overnight recording, *n* = 46; inadequate recording quality, *n* = 2), resulting in a final analytic cohort of 153 patients.

For the present study, we identified 201 patients who underwent first-time catheter ablation for AF and had overnight polygraphy with simultaneous PTT-based BP monitoring during the index hospitalization. Patients were excluded if sinus rhythm was not maintained during the overnight examination or if the recording quality was insufficient for reliable estimation of beat-to-beat BP. After these exclusions, 153 patients were included in the final analytic cohort. The patient selection and exclusion process is summarized in Figure 1.

PTT-derived beat-to-beat BP values were calculated for the present analysis using nighttime recording data that had been obtained and stored as part of routine clinical care. In contrast, AF recurrence and other clinical outcomes were assessed based on medical records and follow-up data. Accordingly, this study represents a *post hoc*, exploratory analysis using registry data collected in routine clinical practice.

Baseline clinical characteristics, including demographic data, comorbidities, medications, laboratory findings, and transthoracic echocardiographic parameters, were obtained from medical records at the baseline outpatient visit within 30 days prior to ablation or at hospital admission, and supplemented by review of electronic medical records when necessary. SDB-related indices, including the apnea–hypopnea index, oxygen desaturation index, mean oxygen saturation, and nadir oxygen saturation, were derived from overnight polygraphy.

This institutional registry study was approved by the Research Ethics Committee of Fukushima Medical University (approval No. 823). Participation in the registry was voluntary, and written informed consent was obtained from all patients at the time of enrollment. The study was conducted in accordance with the principles of the Declaration of Helsinki (24).

### BP monitoring and PTT-based BPV measurement

Overnight sleep studies were performed using a type 3 polygraphy system (SOMNOtouch RESP, Fukuda Denshi Co., Ltd., Tokyo, Japan) synchronized with electrocardiography to derive PTT (18, 25). PTT was defined as the interval from the electrocardiographic R-wave to the upstroke of the fingertip photoplethysmographic pulse (17). Beat-to-beat BP was estimated from PTT using DOMINO Light software version 1.5.0 (Somnomedics, Randersacker, Germany). This software applies a proprietary algorithm (US 2006/0217616 A1; 7,374,542) for continuous recording, as previously described (17).

The nighttime period was defined as 10:00 p.m. to 6:00 a.m. Calibration was performed using a manual brachial cuff BP measurement obtained in the supine position between 3:30 and 4:30 PM on the same day. For each participant, all beat-to-beat systolic blood pressure (SBP), diastolic blood pressure (DBP), and mean blood pressure (MBP) values recorded during the nighttime period were extracted for analysis. From these continuous data, the overall mean, maximum, and minimum values were calculated. Very short-term BPV was quantified as the standard deviation (SD) of all beat-to-beat SBP, DBP, and MBP measurements, during the nighttime period.

### Ablation procedure

All procedures were performed under conscious sedation or general anesthesia. Surface electrocardiograms and intracardiac electrograms were continuously monitored and recorded. After obtaining vascular access, an initial bolus of 3,000 U of heparin was administered. Anticoagulation was subsequently continued with additional infusions and boluses to maintain the activated clotting time between 300 and 400 s throughout the procedure.

The primary ablation strategy was pulmonary vein isolation, performed using either a cryoballoon (Arctic Front Advance; Medtronic, Inc., Minneapolis, MN, USA or POLARx FIT; Boston Scientific, St. Paul, MN, USA) or an irrigated-tip radiofrequency (RF) catheter (SmartTouch Surround Flow; Biosense Webster, Diamond Bar, CA, USA) guided by a 3D electroanatomic mapping system (CARTO3, Biosense Webster). Additional ablation procedures beyond pulmonary vein isolation (e.g., isolation of non-pulmonary vein triggers such as the superior vena cava, or substrate modification) were left to the discretion of the operator.

### Follow-up for AF recurrence

Following the index procedure, the patients were followed for 12 months with outpatient visits scheduled every 1–3 months. Each visit included a clinical assessment of arrhythmia-related symptoms and a 12-lead electrocardiogram. In addition, 24-h Holter monitoring was systematically performed at 3, 6, and 12 months after ablation, as well as whenever patients reported symptoms suggestive of arrhythmia.

The primary endpoint was the first documented recurrence of AF, detected by any electrocardiographic monitoring after a 3-month blanking period, in accordance with the current consensus guidelines (26). Patients were censored at the first documented recurrence or at the end of the 12-month follow-up period if no recurrence was observed.

### Statistical analysis

The normality of continuous variables was assessed using the Shapiro–Wilk test. Normally distributed variables were compared using Student's *t* test, and non-normally distributed variables were compared using the Mann–Whitney *U* test. Categorical variables were analyzed using the chi-square test or Fisher's exact test, as appropriate.

The SDs of SBP, DBP, and MBP were dichotomized into low and high groups according to the median values. AF recurrence–free survival was estimated using the Kaplan–Meier method, and intergroup differences were evaluated using the log-rank test.

To explore factors associated with AF recurrence, univariable Cox proportional hazards models were constructed for baseline clinical variables as well as nighttime PTT-based BP parameters, including SBP, DBP, MBP, and very short-term BPV. Hazard ratios (HRs) and 95% confidence intervals (CIs) were calculated for each parameter. Multivariable analysis was not performed due to the limited number of recurrence events relative to the number of potential covariates.

All analyses were two-sided, and a *P* value < 0.05 was considered statistically significant for group comparisons. Statistical analyses were performed using IBM SPSS Statistics version 29.0 (IBM Corp., Armonk, NY, USA).

## Results

### Baseline characteristics

Table 1 summarizes baseline characteristics for the overall cohort and compares characteristics between the low and high MBP variability groups. The median age was 65 years, and 106 patients (69%) were male. Paroxysmal AF and persistent AF were present in 129 (84%) and 24 (16%) patients, respectively. RF ablation was performed in 91 patients (59%) and cryoballoon ablation in 62 patients (41%); none of the cryoballoon cases required additional RF touch-up. The distribution of ablation modality did not differ significantly between the low and high MBP variability groups. The prevalence of hypertension, dyslipidemia, and diabetes mellitus was 52%, 46%, and 13%, respectively. The mean left atrial diameter was 40.5 mm, and the median left ventricular ejection fraction was 63%. No significant differences in baseline characteristics were observed between the groups. Baseline characteristics stratified by SBP and DBP variability are shown in Supplementary Tables 1, 2.

**Table 1:** Baseline characteristics stratified by MBP variability.

Variables	Total (*n* = 153)	High MBP variability (*n* = 75)	Low MBP variability (*n* = 78)	*P* value
Age, years	65.0 [57.0–71.0]	66.0 [60.0–73.0]	64.0 [55.0–70.0]	0.139
Male, *n* (%)	106 (69)	53 (71)	53 (68)	0.716
Body mass index, kg/m^2^	23.3 [21.7–26.1]	23.8 [21.9–26.7]	22.9 [21.6–25.6]	0.155
Atrial fibrillation type				
Paroxysmal, *n* (%)	129 (84)	64 (85)	65 (83)	0.734
Persistent, *n* (%)	24 (16)	11 (15)	13 (17)	
Ablation modality				
Radiofrequency, *n* (%)	91 (59)	41 (55)	50 (64)	0.235
Cryoballoon, *n* (%)	62 (41)	34 (45)	28 (36)	
Comorbidities				
Hypertension, *n* (%)	79 (52)	39 (52)	40 (51)	0.929
Diabetes, *n* (%)	20 (13)	13 (17)	7 (9)	0.125
Dyslipidemia, *n* (%)	70 (46)	33 (44)	37 (47)	0.670
Smoking, *n* (%)	80 (52)	38 (51)	42 (54)	0.694
Laboratory and echocardiographic data				
B-type natriuretic peptide, pg/mL	34.0 [16.6–90.3]	33.0 [16.7–92.3]	36.7 [16.4–89.6]	0.808
Left atrial diameter, mm	40.5 ± 7.2	41.0 ± 6.5	40.1 ± 7.8	0.413
Left atrial volume index, mL/m^2^	41.0 [35.0–52.5]	41.0 [34.5–48.8]	39.0 [32.5–47.0]	0.450
Left ventricular ejection fraction, %	63.0 [59.0–66.7]	63.0 [57.0–66.0]	64.0 [60.0–67.1]	0.100
Medication				
Beta blockers, *n* (%)	80 (52)	40 (53)	40 (51)	0.800
Class Ⅰ antiarrhythmic drugs, *n* (%)	57 (37)	29 (39)	28 (36)	0.723
Amiodarone, *n* (%)	20 (13)	8 (11)	12 (15)	0.387
Bepridil, *n* (%)	48 (31)	23 (31)	25 (32)	0.854
RAS inhibitors, *n* (%)	67 (44)	35 (47)	32 (41)	0.482
MRAs, *n* (%)	15 (10)	7 (9)	8 (10)	0.848
Calcium channel blockers, *n* (%)	54 (35)	25 (33)	29 (37)	0.619
Loop diuretics, *n* (%)	24 (16)	9 (12)	15 (19)	0.219
SGLT2 inhibitors, *n* (%)	17 (11)	8 (11)	9 (12)	0.864

Values are reported as mean ± standard deviation, median [25th–75th percentile], or number of patients (%). MBP, mean blood pressure; RAS, renin-angiotensin-system; MRA, mineralocorticoid receptor antagonist; SGLT2, sodium-glucose cotransporter 2.

### Nighttime BP and polygraphy findings

Table 2 summarizes nighttime PTT-based BP and sleep study results for the overall cohort and compares characteristics between the low and high MBP variability groups. The mean nighttime MBP was 89.5 ± 11.7 mmHg, and the median SD of MBP was 3.1 [2.5–3.6] mmHg. As expected, the SD of MBP was significantly higher in the high-variability group. The median apnea–hypopnea index was 12.5 [7.4–20.8] events/h, and the median oxygen desaturation index was 11.5 [7.0–20.0] events/h. Sleep study parameters were comparable between the low and high MBP variability groups. Results for SBP and DBP variability were directionally consistent with those for MBP variability and are presented in Supplementary Tables 3, 4.

**Table 2:** Nighttime PTT-based blood pressure and sleep study results stratified by MBP variability.

Variables	Total (*n* = 153)	High variability of MBP (*n* = 75)	Low variability of MBP (*n* = 78)	*P* value
Maximum PTT-based SBP, mmHg	142.2 ± 20.5	147.1 ± 20.5	139.1 ± 16.8	0.010
Minimum PTT-based SBP, mmHg	107.4 ± 17.1	106.7 ± 17.1	109.6 ± 16.8	0.289
Average PTT-based SBP, mmHg	121.4 ± 17.1	123.3 ± 17.7	121.6 ± 16.1	0.553
Standard deviation of PTT-based SBP, mmHg	4.4 [3.7–5.3]	5.3 [4.5–6.0]	3.8 [3.2–4.2]	<0.001
Maximum PTT-based DBP, mmHg	83.9 ± 11.3	84.2 ± 12.3	83.0 ± 10.4	0.539
Minimum PTT-based DBP, mmHg	63.2 ± 12.0	60.2 ± 12.5	65.3 ± 11.0	0.008
Average PTT-based DBP, mmHg	73.6 ± 11.0	72.8 ± 12.0	74.2 ± 10.3	0.437
Standard deviation of PTT-based DBP, mmHg	2.7 [2.3–3.3]	3.3 [2.9–3.9]	2.3 [2.0–2.5]	<0.001
Maximum PTT-based MBP, mmHg	101.9 ± 12.9	103.8 ± 13.6	100.3 ± 11.1	0.085
Minimum PTT-based MBP, mmHg	78.3 ± 12.3	76.0 ± 12.4	80.4 ± 11.6	0.026
Average PTT-based MBP, mmHg	89.5 ± 11.7	89.6 ± 12.5	90.0 ± 11.0	0.818
Standard deviation of PTT-based MBP, mmHg	3.1 [2.5–3.6]	3.6 [3.3–4.3]	2.5 [2.3–2.8]	<0.001
Apnea-hypopnea index, events/hour	12.5 [7.4–20.8]	11.7 [6.3–20.8]	12.6 [7.9–20.4]	0.784
Obstructive apnea index, events/hour	2.2 [0.4–5.1]	2.5 [0.4–5.0]	2.1 [0.5–5.2]	0.822
Central apnea index, events/hour	0.6 [0.1–2.1]	0.5 [0.1–2.4]	0.7 [0.1–1.4]	0.734
Mixed apnea index, events/hour	0.1 [0.0–0.7]	0.1 [0.0–0.4]	0.1 [0.0–0.8]	0.607
Hypopnea index, events/hour	6.9 [4.1–11.2]	6.5 [3.9–12.0]	8.0 [4.2–10.9]	0.547
3% oxygen desaturation index, events/hour	11.5 [7.0–20.0]	11.3 [5.9–20.0]	11.8 [7.6–19.5]	0.815
Percent of sleep time with oxygen desaturation, %	11.3 [6.3–17.0]	11.3 [5.7–19.6]	11.4 [7.5–16.7]	0.940
Average SpO_2_, %	94.0 [93.0–95.0]	94.0 [93.0–95.0]	95.0 [93.0–95.0]	0.618
Lowest SpO_2_, %	86.0 [81.0–88.0]	85.0 [80.0–88.0]	86.0 [81.0–88.0]	0.738

Values are reported as mean ± standard deviation or median [25th–75th percentile]. PTT, pulse transit time; SBP, systolic blood pressure; DBP, diastolic blood pressure; MBP, mean blood pressure.

### AF recurrence

**Figure 2:**
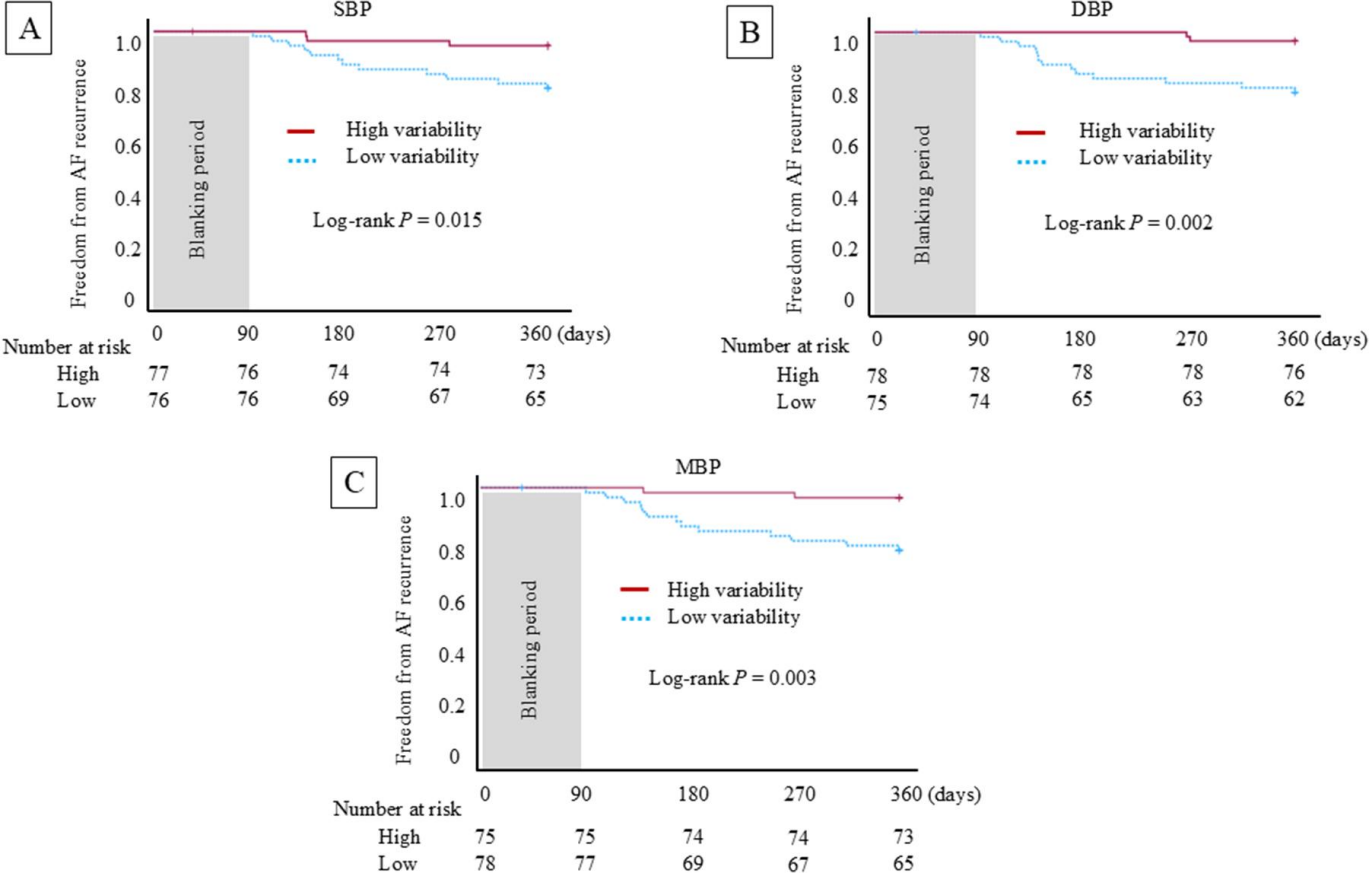
Kaplan–Meier curves for freedom from atrial fibrillation recurrence stratified by median nighttime pulse transit time-based blood pressure variability. Patients were dichotomized according to the median standard deviation of nighttime blood pressure. The higher SBP variability (**A**), DBP variability (**B**), and MBP variability (**C**) showed a significantly lower atrial fibrillation recurrence rate during 12 months of follow-up (SBP variability: log-rank *P* = 0.015; DBP variability: log-rank *P* = 0.002; MBP variability: log-rank *P* = 0.003).

During the 12-month follow-up, AF recurrence occurred in 15 patients (9.8%). Kaplan–Meier analysis demonstrated significantly lower recurrence rates in patients with higher nighttime SBP, DBP, and MBP variability (Figure 2; SBP variability: log-rank *P* = 0.015; DBP variability: *P* = 0.002; and MBP variability: *P* = 0.003).

In univariable Cox proportional hazards analyses, conventional clinical variables, such as sex, AF type, comorbidities, or echocardiographic measurements, were not significantly associated with AF recurrence (Supplementary Tables 5, 6). In contrast, higher SBP, DBP, and MBP variability (SBP variability: HR 0.519, 95% CI 0.311–0.865; DBP variability: HR 0.258, 95% CI 0.101–0.664; MBP variability: HR 0.251, 95% CI 0.099–0.638) were significantly associated with a lower risk of AF recurrence (Table 3).

**Table 3:** Association between nighttime PTT-based BP measures and AF recurrence.

Variables	Hazard ratio	95% confidence interval
Maximum PTT-based SBP, mmHg	0.982	0.956–1.009
Minimum PTT-based SBP, mmHg	0.995	0.965–1.025
Average PTT-based SBP, mmHg	0.988	0.959–1.018
Standard deviation of PTT-based SBP, mmHg	0.519	0.311–0.865
Maximum PTT-based DBP, mmHg	0.970	0.926–1.107
Minimum PTT-based DBP, mmHg	0.999	0.958–1.041
Average PTT-based DBP, mmHg	0.989	0.945–1.035
Standard deviation of PTT-based DBP, mmHg	0.258	0.101–0.664
Maximum PTT-based MBP, mmHg	0.972	0.933–1.013
Minimum PTT-based MBP, mmHg	0.997	0.956–1.038
Average PTT-based MBP, mmHg	0.986	0.945–1.029
Standard deviation of PTT-based MBP, mmHg	0.251	0.099–0.638

PTT, pulse transit time; AF, atrial fibrillation; SBP, systolic blood pressure; DBP, diastolic blood pressure; MBP, mean blood pressure.

## Discussion

This study demonstrated that higher very short-term BPV, assessed through nighttime PTT-based beat-to-beat monitoring, was significantly associated with a lower risk of AF recurrence after first-time ablation. In contrast to previous reports linking higher short- to long-term BPV with incident AF or post-ablation recurrence, our findings indicate an inverse association between very short-term BPV and post-ablation AF recurrence in this specific clinical setting.

A plausible explanation involves baroreflex sensitivity (BRS), which contributes to beat-to-beat BP adjustments through dynamic changes in heart rate and vascular tone (27). When BRS is impaired, autonomic adjustments may become insufficient, potentially resulting in reduced beat-to-beat BPV, that is, lower very short-term BPV. Both impaired BRS and abrupt shifts from sympathetic to parasympathetic predominance immediately before AF onset have been reported in patients with AF (28, 29). In this context, our finding that higher nighttime very short-term BPV was associated with a lower rate of post-ablation AF recurrence may reflect preserved short-term autonomic responsiveness.

Catheter ablation primarily targets pulmonary vein-related arrhythmogenic substrates, but it does not necessarily restore autonomic regulation or reverse advanced atrial remodeling. Autonomic dysfunction, potentially related to reduced BRS, may persist even after technically successful ablation and may be associated with AF recurrence. In addition, patients with more advanced atrial remodeling, reflected by larger left atrial size or impaired atrial function, may exhibit limited reverse remodeling despite ablation. Accordingly, the lower very short-term BPV observed in this study may reflect a combination of blunted baroreflex-driven autonomic adjustments and structural remodeling, which may be associated with AF recurrence. In contrast, higher very short-term BPV may represent an indirect indicator of relatively preserved autonomic function rather than hemodynamic lability.

BPV can be classified by the time scale of assessment: very short-term (beat-to-beat fluctuations over seconds to minutes), short-term (variability within 24 h), mid-term (day-to-day variability), and long-term (visit-to-visit variability) (30). Prior studies have shown that higher short- to long-term BPV is associated with higher risk of incident AF or post-ablation recurrence (13–16). In contrast, the present study found that higher very short-term BPV was associated with a lower risk of post-ablation recurrence. This apparent discrepancy may reflect differences in the mechanisms that govern BPV across time scales. Very short-term BPV has been reported to be influenced by BRS and immediate autonomic regulation (27), and therefore higher variability may be related to relatively preserved autonomic regulation. By comparison, short- to long-term BPV is influenced by more chronic factors, such as lifestyle, medication adherence, sustained sympathetic activity, and vascular remodeling, and higher variability has often been linked to adverse outcomes (13–16). Thus, although reduced autonomic regulation may be a common substrate, BPV may manifest as either lower or higher variability depending on the time scale and measurement modality employed.

We previously reported that increased very short-term BPV, assessed using PTT, was associated with a higher risk of cardiac events in patients with heart failure (18–20). In contrast, the present study demonstrated that higher very short-term BPV was associated with a lower risk of AF recurrence after ablation. This seemingly paradoxical finding may reflect differences in underlying pathophysiology. In heart failure, elevated BPV has been suggested to reflect hemodynamic instability or increased vascular stress, whereas in AF, higher BPV may be related to relatively preserved BRS and autonomic regulation. Accordingly, the clinical significance of very short-term BPV appears to depend on the disease context: elevated variability may be associated with adverse outcomes in heart failure, while higher variability may be associated with preserved autonomic regulation and a lower risk of recurrence in AF. Conventional risk factors for AF recurrence, including age, left atrial diameter, hypertension, and diabetes (8, 11), were not significantly associated with recurrence in the present study. Several factors may support this finding, such as the relatively homogeneous population limited to first-time ablation cases, the small number of recurrence events, and the standardized perioperative management protocol. In contrast, the observed association between higher very short-term BPV and lower AF recurrence suggests that preserved autonomic regulation, which is difficult to capture using conventional clinical indicators, may be relevant for risk stratification after ablation.

Assessment of very short-term BPV using PTT may provide a noninvasive and cuffless approach for evaluating beat-to-beat autonomic regulation, which is not reflected in conventional cuff-measured BP. This information may help inform therapeutic strategies after AF ablation, such as more intensive rhythm monitoring during the blanking period or pharmacological and lifestyle interventions aimed at improving autonomic balance, potentially contributing to more individualized postoperative management and improving long-term outcomes.

## Study limitations

This study has several limitations. First, it was a single-center study with a relatively small sample size and a limited number of recurrence events, which may have reduced statistical power. Given the limited number of recurrence events, multivariable adjustment was not performed to avoid model overfitting; therefore, residual confounding cannot be excluded. In addition, the hazard ratio estimates had wide confidence intervals, and the findings should be interpreted as exploratory (pilot) and hypothesis-generating. Second, PTT-based measurements depended on cuff calibration and a proprietary algorithm, and potential measurement errors cannot be entirely excluded. Although the PTT-based system used in this study is based on a previously evaluated algorithm for cuffless BP estimation, formal validation specifically for beat-to-beat BPV assessment and in AF populations remains limited. Importantly, BP measurement and BPV analyses in the present study were performed during sinus rhythm, which may have minimized rhythm-related measurement uncertainty. Third, the inclusion of both RF and cryoballoon ablation may have introduced heterogeneity. Because these techniques can differ in autonomic modulation, this variability could have influenced recurrence rates. Fourth, direct assessment of BRS was not performed, leaving significant uncertainty in the mechanistic interpretation. Fifth, daytime data were unavailable, preventing the evaluation of 24-h BPV or nocturnal dipping patterns. Sixth, since the patients in this study had only mild-to-moderate SDB as defined by the apnea–hypopnea index, the association between more severe SDB and AF recurrence remains unclear. Finally, because beat-to-beat BPV assessment required sinus rhythm during the overnight examination, patients with persistent AF were less likely to meet inclusion criteria, which may have contributed to the predominance of paroxysmal AF in the analytic cohort. This retrospective analysis included only patients who underwent overnight polygraphy with simultaneous PTT-based beat-to-beat BP monitoring. Therefore, the generalizability of our findings to broader AF ablation populations (e.g., persistent AF) may be limited; differences according to AF type should be confirmed in larger studies.

## Conclusion

This study demonstrated that higher nighttime very short-term BPV was associated with a lower risk of AF recurrence after ablation, which may be related to preserved autonomic regulation. These findings suggest that PTT-based nighttime BP monitoring may provide a potentially practical and noninvasive approach for characterizing the risk of AF recurrence after ablation. Further validation through direct comparison with BRS indices and larger multicenter studies is warranted to confirm the generalizability and clinical applicability of these findings.

## Data Availability

The raw data supporting the conclusions of this article will be made available by the authors, without undue reservation.
