# Supplementary material for: Higher very short-term blood pressure variability is associated with lower atrial fibrillation recurrence after catheter ablation
Source: Front Cardiovasc Med. 2026 Mar 16;13:1779540. doi: 10.3389/fcvm.2026.1779540 (PMC13033510; doi:10.3389/fcvm.2026.1779540)
Supplement: Supplementary file 1 [file Table1.docx]

**Supplementary Table 1**. Baseline characteristics stratified by SBP variability.

|  | **Total**  **(*n* = 153)** | **High SBP variability**  **(*n* = 77)** | **Low SBP**  **variability**  **(*n* = 76)** | ***P* value** |
| --- | --- | --- | --- | --- |
| Age, years | 65.0 [57.0–71.0] | 64.0 [57.0–73.0] | 65.5 [57.3–70.0] | 0.937 |
| Male, *n* (%) | 106 (69) | 54 (70) | 52 (68) | 0.819 |
| Body mass index, kg/m^2^ | 23.3 [21.7–26.1] | 23.9 [21.9–26.7] | 23.2 [21.5–25.6] | 0.125 |
| Atrial fibrillation type  Paroxysmal, *n* (%)  Persistent, *n* (%) | 129 (84)  24 (16) | 64 (83)  13 (17) | 65 (86)  11 (14) | 0.682 |
| Ablation modality  　Radiofrequency, *n* (%)  Cryoballoon, *n* (%) | 91 (59)  62 (41) | 44 (57)  33 (43) | 47 (62)  29 (38) | 0.554 |
| **Comorbidities** |  |  |  |  |
| Hypertension, *n* (%) | 79 (52) | 44 (57) | 35 (46) | 0.170 |
| Diabetes, *n* (%) | 20 (13) | 10 (13) | 10 (13) | 0.975 |
| Dyslipidemia, *n* (%) | 70 (46) | 37 (48) | 33 (43) | 0.565 |
| Smoking, *n* (%) | 80 (52) | 41 (53) | 39 (51) | 0.811 |
| **Laboratory and echocardiographic data** |  |  |  |  |
| B-type natriuretic peptide, pg/mL | 34.0 [16.6–90.3] | 30.2 [15.8–63.8] | 38.9 [16.8–119.6] | 0.167 |
| Left atrial diameter, mm | 40.5 ± 7.2 | 40.2 ± 6.5 | 40.8 ± 7.9 | 0.608 |
| Left atrial volume index, mL/m^2^ | 40.4 [33.2–47.0] | 40.0 [33.5–45.8] | 42.0 [33.0–50.0] | 0.490 |
| Left ventricular ejection fraction, % | 63.0 [59.0–66.7] | 64.0 [60.0–68.0] | 63.0 [58.3–65.9] | 0.067 |
| **Medication** |  |  |  |  |
| Beta blockers, *n* (%) | 80 (52) | 35 (45) | 45 (59) | 0.089 |
| Class Ⅰ antiarrhythmic drugs, *n* (%) | 57 (37) | 32 (42) | 25 (33) | 0.268 |
| Amiodarone, *n* (%) | 20 (13) | 5 (6) | 15 (20) | 0.015 |
| Bepridil, n (%) | 48 (31) | 28 (36) | 20 (26) | 0.180 |
| RAS inhibitors, *n* (%) | 67 (44) | 34 (44) | 33 (43) | 0.927 |
| MRAs, *n* (%) | 15 (10) | 3 (4) | 12 (16) | 0.013 |
| Calcium channel blockers, *n* (%) | 54 (35) | 32 (42) | 22 (29) | 0.103 |
| Loop diuretics, *n* (%) | 24 (16) | 7 (9) | 17 (22) | 0.024 |
| SGLT2 inhibitors, *n* (%) | 17 (11) | 13 (17) | 4 (5) | 0.019 |

Values are reported as mean ± standard deviation, median [25th–75th percentile], or number of patients (%). SBP, systolic blood pressure; RAS, renin-angiotensin-system; MRA, mineralocorticoid receptor antagonist; SGLT2, sodium-glucose cotransporter 2.
