# Supplementary material for: Higher very short-term blood pressure variability is associated with lower atrial fibrillation recurrence after catheter ablation
Source: Front Cardiovasc Med. 2026 Mar 16;13:1779540. doi: 10.3389/fcvm.2026.1779540 (PMC13033510; doi:10.3389/fcvm.2026.1779540)
Supplement: Supplementary file 2 [file Table2.docx]

**Supplementary Table 2**. Baseline characteristics stratified by DBP variability.

|  | **Total**  **(*n* = 153)** | **High DBP variability**  **(*n* = 78)** | **Low DBP variability**  **(*n* = 75)** | ***P* value** |
| --- | --- | --- | --- | --- |
| Age, years | 65.0 [57.0–71.0] | 66.5 [60.0–73.3] | 63.0 [56.0–69.0] | 0.937 |
| Male, *n* (%) | 106 (69) | 53 (68) | 53 (71) | 0.716 |
| Body mass index, kg/m^2^ | 23.3 [21.7–26.1] | 23.9 [21.9–26.6] | 22.9 [21.5–25.6] | 0.125 |
| Atrial fibrillation type  Paroxysmal, *n* (%)  Persistent, *n* (%) | 129 (84)  24 (16) | 65 (83)  13 (17) | 64 (85)  11 (15) | 0.734 |
| Ablation modality  　Radiofrequency, *n* (%)  Cryoballoon, *n* (%) | 91 (59)  62 (41) | 45 (58)  33 (42) | 46 (61)  29 (39) | 0.647 |
| **Comorbidities** |  |  |  |  |
| Hypertension, *n* (%) | 79 (52) | 41 (53) | 38 (51) | 0.814 |
| Diabetes, *n* (%) | 20 (13) | 15 (19) | 5 (7) | 0.021 |
| Dyslipidemia, *n* (%) | 70 (46) | 33 (42) | 37 (49) | 0.383 |
| Smoking, *n* (%) | 80 (52) | 36 (46) | 44 (59) | 0.121 |
| **Laboratory and echocardiographic data** |  |  |  |  |
| B-type natriuretic peptide, pg/mL | 34.0 [16.6–90.3] | 41.2 [17.8–120.4] | 29.3 [16.2–65.5] | 0.167 |
| Left atrial diameter, mm | 40.5 ± 7.2 | 41.6 ± 6.6 | 39.5 ± 7.7 | 0.072 |
| Left atrial volume index, mL/m^2^ | 41.0 [35.0–52.5] | 39.0 [32.5–46.0] | 41.0 [35.0–52.5] | 0.490 |
| Left ventricular ejection fraction, % | 63.0 [59.0–66.7] | 65.0 [60.0–68.0] | 61.7 [57.0–65.0] | 0.067 |
| **Medication** |  |  |  |  |
| Beta blockers, *n* (%) | 80 (52) | 48 (62) | 32 (43) | 0.019 |
| Class Ⅰ antiarrhythmic drugs, *n* (%) | 57 (37) | 27 (35) | 30 (40) | 0.491 |
| Amiodarone, *n* (%) | 20 (13) | 13 (17) | 7 (9) | 0.179 |
| Bepridil, n (%) | 48 (31) | 23 (29) | 25 (33) | 0.608 |
| RAS inhibitors, *n* (%) | 67 (44) | 40 (51) | 27 (36) | 0.057 |
| MRAs, *n* (%) | 15 (10) | 10 (13) | 5 (7) | 0.201 |
| Calcium channel blockers, *n* (%) | 54 (35) | 27 (35) | 27 (36) | 0.858 |
| Loop diuretics, *n* (%) | 24 (16) | 12 (15) | 12 (16) | 0.917 |
| SGLT2 inhibitors, *n* (%) | 17 (11) | 10 (13) | 7 (9) | 0.493 |

Values are reported as mean ± standard deviation, median [25th–75th percentile], or number of patients (%). DBP, diastolic blood pressure; RAS, renin-angiotensin-system; MRA, mineralocorticoid receptor antagonist; SGLT2, sodium-glucose cotransporter 2.
