# Supplementary material for: Higher very short-term blood pressure variability is associated with lower atrial fibrillation recurrence after catheter ablation
Source: Front Cardiovasc Med. 2026 Mar 16;13:1779540. doi: 10.3389/fcvm.2026.1779540 (PMC13033510; doi:10.3389/fcvm.2026.1779540)
Supplement: Supplementary file 3 [file Table3.docx]

**Supplementary Table 3.** Nighttime PTT-based blood pressure and sleep study results stratified by SBP variability.

|  | **Total (*n* = 153)** | **High variability of SBP (*n* = 77)** | **Low variability of SBP (n = 76)** | ***P* value** |
| --- | --- | --- | --- | --- |
| Maximum PTT-based SBP, mmHg | 142.2 ± 20.5 | 148.5 ± 17.7 | 137.4 ± 19.4 | < 0.001 |
| Minimum PTT-based SBP, mmHg | 107.4 ± 17.1 | 106.9 ± 14.4 | 109.5 ± 19.3 | 0.357 |
| Average PTT-based SBP, mmHg | 121.4 ± 17.1 | 124.0 ± 14.7 | 120.9 ± 18.8 | 0.258 |
| Standard deviation of PTT-based SBP, mmHg | 4.4 [3.7–5.3] | 5.3 [4.8–6.0] | 3.7 [3.2–4.0] | < 0.001 |
| Maximum PTT-based DBP, mmHg | 83.9 ± 11.3 | 84.1 ± 11.3 | 83.0 ± 11.5 | 0.559 |
| Minimum PTT-based DBP, mmHg | 63.2 ± 12.0 | 62.0 ± 11.3 | 63.5 ± 12.7 | 0.430 |
| Average PTT-based DBP, mmHg | 73.6 ± 11.0 | 73.5 ± 10.6 | 73.5 ± 11.7 | 0.986 |
| Standard deviation of PTT-based DBP, mmHg | 2.7 [2.3–3.3] | 3.0 [2.5–3.8] | 2.5 [2.0–2.9] | < 0.001 |
| Maximum PTT-based MBP, mmHg | 101.9 ± 12.9 | 104.1 ± 11.7 | 99.8 ± 12.9 | 0.033 |
| Minimum PTT-based MBP, mmHg | 78.3 ± 12.3 | 77.3 ± 10.4 | 79.2 ± 13.7 | 0.353 |
| Average PTT-based MBP, mmHg | 89.5 ± 11.7 | 90.4 ± 10.4 | 89.0 ± 13.0 | 0.577 |
| Standard deviation of PTT-based MBP, mmHg | 3.1 [2.5–3.6] | 3.6 [3.2–4.3] | 2.6 [2.2–2.9] | < 0.001 |
| Apnea-hypopnea index, events/hour | 12.5 [7.4–20.8] | 11.7 [6.6–20.8] | 12.6 [7.7–20.7] | 0.758 |
| Obstructive apnea index, events/hour | 2.2 [0.4–5.1] | 1.8 [0.4–5.9] | 2.4 [0.5–5.0] | 0.900 |
| Central apnea index, events/hour | 0.6 [0.1–2.1] | 0.5 [0.1–2.3] | 0.7 [0.2–1.7] | 0.353 |
| Mixed apnea index, events/hour | 0.1 [0.0–0.7] | 0.1 [0.0–0.5] | 0.1 [0.0–0.8] | 0.829 |
| Hypopnea index, events/hour | 6.9 [4.1–11.2] | 6.6 [4.0–11.6] | 7.9 [4.2–11.1] | 0.770 |
| 3% oxygen desaturation index, events/hour | 11.5 [7.0–20.0] | 11.2 [6.2–20.0] | 12.1 [7.6–20.0] | 0.663 |
| Percent of sleep time with oxygen desaturation, % | 11.3 [6.3–17.0] | 11.3 [5.7–17.0] | 11.3 [7.5–18.8] | 0.698 |
| Average SpO_2_, % | 94.0 [93.0–95.0] | 94.0 [93.0–95.0] | 95.0 [93.0–95.0] | 0.315 |
| Lowest SpO_2_, % | 86.0 [81.0–88.0] | 86.0 [81.0–88.0] | 85.0 [80.0–88.0] | 0.853 |

Values are reported as mean ± standard deviation or median [25th–75th percentile]. PTT, pulse transit time; SBP, systolic blood pressure; DBP, diastolic blood pressure; MBP, mean blood pressure.
