# Supplementary material for: Higher very short-term blood pressure variability is associated with lower atrial fibrillation recurrence after catheter ablation
Source: Front Cardiovasc Med. 2026 Mar 16;13:1779540. doi: 10.3389/fcvm.2026.1779540 (PMC13033510; doi:10.3389/fcvm.2026.1779540)
Supplement: Supplementary file 4 [file Table4.docx]

**Supplementary Table 4.** Nighttime PTT-based blood pressure and sleep study results stratified by DBP variability.

|  | **Total (*n* = 153)** | **High variability of DBP (*n* = 78)** | **Low variability of DBP (n = 75)** | ***P* value** |
| --- | --- | --- | --- | --- |
| Maximum PTT-based SBP, mmHg | 142.2 ± 20.5 | 145.1 ± 21.7 | 140.9 ± 16.4 | 0.178 |
| Minimum PTT-based SBP, mmHg | 107.4 ± 17.1 | 108.0 ± 18.3 | 108.4 ± 15.6 | 0.862 |
| Average PTT-based SBP, mmHg | 121.4 ± 17.1 | 123.2 ± 18.9 | 121.6 ± 14.5 | 0.553 |
| Standard deviation of PTT-based SBP, mmHg | 4.4 [3.7–5.3] | 4.9 [4.0–5.9] | 4.0 [3.3–4.6] | < 0.001 |
| Maximum PTT-based DBP, mmHg | 83.9 ± 11.3 | 84.1 ± 11.3 | 83.0 ± 11.5 | 0.163 |
| Minimum PTT-based DBP, mmHg | 63.2 ± 12.0 | 60.4 ± 13.0 | 65.3 ± 10.5 | 0.011 |
| Average PTT-based DBP, mmHg | 73.6 ± 11.0 | 73.3 ± 12.5 | 73.7 ± 9.6 | 0.825 |
| Standard deviation of PTT-based DBP, mmHg | 2.7 [2.3–3.3] | 3.3 [2.9–3.8] | 2.3 [1.9–2.5] | < 0.001 |
| Maximum PTT-based MBP, mmHg | 101.9 ± 12.9 | 103.5 ± 14.1 | 100.4 ± 10.4 | 0.128 |
| Minimum PTT-based MBP, mmHg | 78.3 ± 12.3 | 76.5 ± 13.1 | 80.1 ± 10.8 | 0.064 |
| Average PTT-based MBP, mmHg | 89.5 ± 11.7 | 89.9 ± 13.1 | 89.7 ± 10.1 | 0.915 |
| Standard deviation of PTT-based MBP, mmHg | 3.1 [2.5–3.6] | 3.6 [3.2–4.3] | 2.5 [2.2–2.9] | < 0.001 |
| Apnea-hypopnea index, events/hour | 12.5 [7.4–20.8] | 12.0 [7.1–20.7] | 12.6 [7.5–20.8] | 0.974 |
| Obstructive apnea index, events/hour | 2.2 [0.4–5.1] | 2.2 [0.4–5.7] | 2.2 [0.4–4.5] | 0.752 |
| Central apnea index, events/hour | 0.6 [0.1–2.1] | 0.5 [0.1–2.3] | 0.6 [0.2–1.4] | 0.589 |
| Mixed apnea index, events/hour | 0.1 [0.0–0.7] | 0.0 [0.0–0.5] | 0.1 [0.0–0.7] | 0.293 |
| Hypopnea index, events/hour | 6.9 [4.1–11.2] | 6.6 [4.0–12.1] | 7.9 [4.2–11.0] | 0.758 |
| 3% oxygen desaturation index, events/hour | 11.5 [7.0–20.0] | 11.7 [6.8–20.0] | 11.5 [7.3–19.3] | 0.882 |
| Percent of sleep time with oxygen desaturation, % | 11.3 [6.3–17.0] | 11.5 [5.9–18.8] | 10.9 [7.3–16.7] | 0.803 |
| Average SpO_2_, % | 94.0 [93.0–95.0] | 94.0 [93.0–95.0] | 95.0 [94.0–95.0] | 0.059 |
| Lowest SpO_2_, % | 86.0 [81.0–88.0] | 85.0 [80.0–88.0] | 86.0 [81.0–88.0] | 0.701 |

Values are reported as mean ± standard deviation or median [25th–75th percentile]. PTT, pulse transit time; DBP, diastolic blood pressure; SBP, systolic blood pressure; MBP, mean blood pressure.
