# Supplementary material for: Higher very short-term blood pressure variability is associated with lower atrial fibrillation recurrence after catheter ablation
Source: Front Cardiovasc Med. 2026 Mar 16;13:1779540. doi: 10.3389/fcvm.2026.1779540 (PMC13033510; doi:10.3389/fcvm.2026.1779540)
Supplement: Supplementary file 5 [file Table5.docx]

**Supplementary Table 5.** Association between baseline characteristics and AF recurrence.

|  | **Hazard ratio** | **95% confidence interval** |
| --- | --- | --- |
| Age | 0.990 | 0.943–1.038 |
| Female | 0.574 | 0.162–2.033 |
| Body mass index | 0.987 | 0.857–1.136 |
| Persistent atrial fibrillation | 0.372 | 0.049–2.831 |
| Cryoballoon ablation | 1.305 | 0.473–3.598 |
| **Comorbidities** |  |  |
| Hypertension | 1.063 | 0.385–2.931 |
| Diabetes | 0.040 | 0.000–23.656 |
| Dyslipidemia | 0.595 | 0.203–1.740 |
| Smoking | 1.879 | 0.642–5.497 |
| **Laboratory and echocardiographic data** |  |  |
| B-type natriuretic peptide | 1.000 | 0.997–1.004 |
| Left atrial diameter | 1.006 | 0.938–1.080 |
| Left atrial volume index | 1.002 | 0.970–1.035 |
| Left ventricular ejection fraction | 1.036 | 0.964–1.112 |
| **Medication** |  |  |
| Beta blockers | 1.373 | 0.489–3.856 |
| Class Ⅰ antiarrhythmic drugs | 1.553 | 0.563–4.284 |
| Amiodarone | 0.040 | 0.000–23.656 |
| Bepridil | 1.435 | 0.511–4.032 |
| RAS inhibitors | 0.853 | 0.304–2.397 |
| MRAs | 0.042 | 0.000–61.709 |
| Calcium channel blockers | 1.701 | 0.617–4.691 |
| Loop diuretics | 0.819 | 0.185–3.629 |
| SGLT2 inhibitors | 0.041 | 0.000–40.032 |

AF, atrial fibrillation; RAS, renin-angiotensin-system; MRA, mineralocorticoid receptor antagonist; SGLT2, sodium-glucose cotransporter 2.
