# Supplementary material for: Higher very short-term blood pressure variability is associated with lower atrial fibrillation recurrence after catheter ablation
Source: Front Cardiovasc Med. 2026 Mar 16;13:1779540. doi: 10.3389/fcvm.2026.1779540 (PMC13033510; doi:10.3389/fcvm.2026.1779540)
Supplement: Supplementary file 6 [file Table6.docx]

**Supplementary Table 6.** Association between nighttime polygraphy parameters and AF recurrence.

|  | **Hazard ratio** | **95% confidence interval** |
| --- | --- | --- |
| Apnea–hypopnea index | 1.007 | 0.966–1.051 |
| Obstructive apnea index | 0.974 | 0.866–1.096 |
| Central apnea index | 0.902 | 0.711–1.144 |
| Mixed apnea index | 0.408 | 0.107–1.551 |
| Hypopnea index | 1.044 | 0.993–1.099 |
| 3% oxygen desaturation index | 1.010 | 0.968–1.054 |
| Percent of sleep time with oxygen desaturation | 1.004 | 0.951–1.059 |
| Mean SpO_2_ | 1.069 | 0.766–1.492 |
| Lowest SpO_2_ | 1.010 | 0.929–1.098 |

AF, atrial fibrillation.
